# Biodiversity Conservation in the REDD

**DOI:** 10.1186/1750-0680-5-7

**Published:** 2010-11-23

**Authors:** Gary D Paoli, Philip L Wells, Erik Meijaard, Matthew J Struebig, Andrew J Marshall, Krystof Obidzinski, Aseng Tan, Andjar Rafiastanto, Betsy Yaap, JW Ferry Slik, Alexandra Morel, Balu Perumal, Niels Wielaard, Simon Husson, Laura D'Arcy

**Affiliations:** 1 Daemeter Consulting, Bogor, Indonesia; 2 People and Nature Consulting International, Jakarta, Indonesia; 3 School of Archaeology and Anthropology, Australian National University, Canberra, Australia; 4 Durrell Institute of Conservation and Ecology, University of Kent, Canterbury, UK; 5 School of Biological & Chemical Sciences, Queen Mary University of London, London, UK; 6 Department of Anthropology, University of California at Davis, Davis, USA; 7 Center for International Forestry and Agricultural Research, Bogor, Indonesia; 8 Fauna and Flora International Indonesia Program, Jakarta, Indonesia; 9 Xishuangbanna Tropical Botanical Garden, Chinese Academy of Sciences, Menglun, China; 10 Environmental Change Institute, University of Oxford, UK; 11 Global Environment Centre, Selangor, Malaysia; 12 SarVision, University of Wageningen, The Netherlands; 13 The Orangutan Tropical Peatland Project, Center for International Cooperation in Tropical Peatlands, Palangkaraya, Indoensia

## Abstract

Deforestation and forest degradation in the tropics is a major source of global greenhouse gas (GHG) emissions. The tropics also harbour more than half the world's threatened species, raising the possibility that reducing GHG emissions by curtailing tropical deforestation could provide substantial co-benefits for biodiversity conservation. Here we explore the potential for such co-benefits in Indonesia, a leading source of GHG emissions from land cover and land use change, and among the most species-rich countries in the world. We show that focal ecosystems for interventions to reduce emissions from deforestation and forest degradation in Indonesia do not coincide with areas supporting the most species-rich communities or highest concentration of threatened species. We argue that inherent trade-offs among ecosystems in emission reduction potential, opportunity cost of foregone development and biodiversity values will require a regulatory framework to balance emission reduction interventions with biodiversity co-benefit targets. We discuss how such a regulatory framework might function, and caution that pursuing emission reduction strategies without such a framework may undermine, not enhance, long-term prospects for biodiversity conservation in the tropics.

## Introduction

Carbon emissions from deforestation and forest degradation contribute 12-20% of anthropogenic global greenhouse gas (GHG) emissions annually [1,2], primarily from the tropics [3]. Tropical countries also harbour over half (51.1%) of the world's 48,170 threatened species [4], raising the possibility that reducing GHG emissions by curtailing tropical deforestation might also provide valuable co-benefits for biodiversity conservation [5]. Here we explore potential biodiversity impacts of anticipated emission reduction strategies in Indonesia, the world's third largest source of GHG emissions [6] and among the most species-rich countries in the world. We address calls in this journal [7,8] and elsewhere [9-11] for a stronger regulatory framework governing emission reduction strategies in forests to ensure that biodiversity co-benefits are achieved. We caution that in Indonesia and other tropical countries, pursuing emission reduction strategies in forests without such a framework may worsen, not enhance, long-term biodiversity conservation.

The Reducing Emissions from forest Degradation and Deforestation (REDD) scheme of the post-Kyoto UN Framework Convention on Climate Change (UNFCCC) treaty seeks to involve developing countries in global GHG reduction efforts by creating financial incentives to improve forest management and protection [12]. Under REDD, and its derivative REDD+, which recognizes forest carbon stock enhancements (sequestration) from improved conservation and sustainable management of forests, developing countries that reduce forest based emissions below an established 'business as usual' projection will be rewarded through payments from donor funds or market sale of emission reduction credits.

REDD clearly provides an opportunity for biodiverse, carbon-rich tropical countries to protect threatened biodiversity as a co-benefit of maintaining forests and the carbon they store [11,13]. However, it remains unclear how biodiversity provisions will be included within REDD, raising questions about the extent to which it will improve biodiversity conservation over the long-term [5,14,15]. Estimated terrestrial carbon and biodiversity are positively correlated globally [11], but this pattern does not necessarily hold at sub-national scales where REDD will typically be implemented. This raises concern that preferential targeting of carbon-rich ecosystems may intensify pressures on relatively carbon-poor ecosystems that nevertheless support equal or greater levels of biodiversity [15-17].

## Discussion

### REDD in Indonesia

Indonesia, where REDD will be pursued as a set of sub-national programs, illustrates the need for explicit biodiversity provisions to ensure that biodiversity co-benefits are achieved, and unanticipated negative outcomes are avoided.

Indonesia is a rapidly growing developing country, with economic growth of 4.5-6.3% annually over the past three years [18] due in part to expanding natural resource industries such as oil palm, fiber plantations and pulp. Continued growth of these sectors is central to government plans to expand exports and create jobs. The Ministry of Forestry recently announced a 10-year plan to develop nine million ha of fiber plantations to supply a two-fold expansion of pulp and paper capacity [19]. Similarly, up to 10 million ha of new oil palm plantations are projected for development by 2020 to meet growing demand for palm oil derived products [20,21]. Together, these industries will require an estimated 19 million ha of land for new plantations over the next 10 years.

Plantation expansion notwithstanding, Indonesia has also made voluntary commitments to reduce emissions by 26% by 2020, or up to 41% if financial support is forthcoming from the international community [22]. Such commitments have drawn significant attention, including a recent offer from Norway of US$1 billion to Indonesia for assistance with implementing REDD [23], and up to 45 REDD projects under development as of early 2010 [24,25].

### Sources of forest based emissions in Indonesia

Approximately 85% of Indonesia's estimated 3.01 Gt CO_2 _annual emissions in 2005 originated from deforestation and degradation [6]. The main sources of these emissions are lowland dipterocarp forests on well-drained mineral soils and peat swamp forest on water-logged peatlands, with estimated original extent of c. 128.1 million ha and c. 20.1 million ha, respectively (Table 1). Estimated aboveground carbon is similar in forests on mineral soils and peat (211 ± 55 vs. 230 ± 66 t C ha^-1^, respectively, mean ± SD; Table 1, see Additional File 1: Datafile_1.xls for original data). However, belowground carbon stocks differ markedly, with up to c. 20 times more carbon in the un-decomposed organic matter of peat compared to mineral soils (137 ± 26 vs 2425 ± 726 t C ha^-1^; Table 1). Total carbon stocks are thus, on average, eight times higher in lowland forests on peat than on mineral soils, with corresponding higher total estimated GHG emissions arising from their conversion (Table 1).

**Table 1:** Physical attributes and emission estimates for lowland tropical forest (<500 m a.s.l.) on peat and mineral substrates in Indonesia

	Lowland forest on contrasting substrates	
	
Attribute	Mineral soils	Peat
*(a) Estimated original extent in Indonesia *(1000 ha) ^a^	128,100	20,949

*(b) Carbon Stocks *(mean ± sd; range)		

Aboveground stocks (t C ha^-1^) ^b^	211 ± 55 (100-370)	230 ± 66 (148-3510)

Belowground stocks (t C ha^-1^)	137 ± 26 (98-168) ^c^	2425 ± 726 (600-3131) ^d^

Total (t C ha^-1^)	353 (214-539)	2680 (748-3496)

*(c) CO*_*2 *_*emissions *(t CO_2 _ha^-1 ^yr^-1^)		

Estimated net annual CO_2 _emissions from oil palm plantations (mean ± sd; range) ^e^	13.7 ± 5.6 (8.1-25.3)	58.6 ± 18.2 (43.7-87.0)

Estimated net annual CO_2 _emissions from fiber plantations ^f^	16.6	48.4

Estimated total annual CO_2 _emissions from deforestation and degradation across Indonesia (range) ^g^	538-1596	2121-4611

*(d) Estimated original extent of lowland forest in Kalimantan and occurrence of remaining forest as of 2008 in different land use classes according to national spatial plans *(percentage of total remaining area in parentheses) ^h^		

Estimated original extent (ha)	39,921,309	4,321,178

Estimated remaining extent as of 2008 (ha)	21,508,044	2,382,313

- Forest allocated for Protection	3,663,783 (17.0)	195,606 (8.2)

- Forest allocated for Production	12,429,890 (57.8)	1,268,977 (53.3)

- Forest allocated for Conversion	5,414,371 (25.2)	917,730 (38.5)

Comparisons are made between (a) estimated original extent of forest on peat and mineral substrates across Indonesia; (b) carbon stocks and (c) emission levels from both Kalimantan and Sumatra; and (d) estimated original and remaining 2008 lowland forest cover in Kalimantan on peat and mineral soil, separated by national land use classification.

^a ^Estimated extent of 2008 forest cover derived from SPOT Veg imagery (1 km^2 ^resolution) by SarVision, overlaid with SRTM to define areas <500 m a.s.l. [26] and Wetlands International map of peat lands to define areas with surface peat >50 cm depth [27-29]. Total extent of lowland forest on mineral soils was estimated as all other forest <500 m a.s.l. not on peat, and includes lowland rain forest on well drained mineral soils (c. 107 of 128 million ha, 84% of the total], as well as forest on limestone, ultrabasic rock, nutrient poor sandy soils on which kerangas (heath) forest develops, mangroves and freshwater swamps.

^b ^See Additional File 1: Datafile_1.xls; [30-49].

^c ^Data from [50].

^d ^Data from [13,51-53].

^e ^See Additional File 1: Datafile_1.xls; [54-58].

^f ^Data from [55].

^g ^Includes emissions originating from deforestation, degradation, peat land decomposition from drainage and fires; data from [59-61].

^h ^Using data for lowland forest on mineral soils described under note (a), the forest area for Kalimantan was further subdivided according to land use status as defined by the Indonesian Ministry of Forestry (Tata Guna Hutan Kesepakatan, TGHK). Protection Forest areas are allocated for conservation purposes and may not be exploited; Production Forest areas may be logged and/or converted to industrial wood fiber plantations but not agriculture; Conversion Forest areas are allocated for planned conversion to non-forest agricultural crops (including e.g. oil palm or rubber).

Historically, deforestation rates on peat were much lower than on mineral soils, reflecting higher costs, lower yield and technological challenges of developing peatlands [62]. From 1985-1997, relative losses of lowland forest on mineral soils in Sumatra and Kalimantan were nearly three times higher than forests on the coastal alluvial plains dominated by peat (61% vs 24% in Sumatra; 58% vs 23% in Kalimantan; data from [63]).

Increased use of technology, however, such as excavators, coupled with expanding trade and rising demand for land have stimulated large-scale drainage of forested peatlands for transmigration projects and agricultural development [64-66]. Drainage and resulting oxidation of carbon-dense peat, combined with annual fires [60,61], made peat the source of nearly half (45%, 1.35 Gt CO_2 _yr^-1^) of Indonesia's annual emissions, and 3% of global emissions, in 2005 (Table 1; [6,67]). Further, destructive synergies with extreme drought linked to El Nino Southern Oscillation increase risk of catastrophic fires, such as the 1997-98 peat land fires in Kalimantan that caused emissions estimated to represent 13-40% of global emissions originating from fossil fuels during that period [68].

### Reconciling plantation expansion with emissions reduction

One option to expand plantations and meet emission reduction targets in Indonesia would be to concentrate new plantations on degraded, deforested land, of which c. 23 million ha in critical condition were mapped across Indonesia in 2006 [69]. Planting such 'degraded lands' has proven to be a challenge, however, due to the scarcity of land meeting an ecologically and socially sound definition of degraded, and the fact that much deforested land is in fact under some form of management by local communities.

Given the much higher total carbon storage (emission reduction potential) of forests on peat (Table 1), and lower opportunity cost of foregoing peatland development, limiting further conversion of peat would seem a preferred means to reconcile economic growth and emissions reduction. Indeed, the Indonesian government recently expressed this view [70]; Norway has made it a pre-condition of its $US1 billion offer [23]; and peatlands, despite their lesser extent than mineral areas (Table 1), have drawn the majority of REDD project investments, with 11 of 17 site-based carbon projects in Sumatra and Kalimantan on peat, equal to 1.69 million (56%) of the estimated 3.06 million ha of REDD projects across Indonesia (see Additional File 2: Datafile_2.pdf).

### Unexpected outcomes for biodiversity

Tropical lowland forests on peat or mineral soils are priority areas for biodiversity conservation, yet are typically underrepresented in protected area networks relative to upland habitats [72]. Greater protection of Indonesian peatlands under REDD therefore would not only achieve emission reductions, but also help conserve a unique ecosystem that supports specialized aquatic and plant biodiversity [73-76], and provides wilderness habitat for some of Indonesia's most endangered large vertebrates, including Sumatran tigers, Asian elephants, orangutan and false gharial [77-80]. Nevertheless, if REDD is implemented with a disproportionate focus on peat, and Indonesia pursues goals for 19 million ha of new plantations over the next 10 years, then the potential for REDD to promote conservation for the majority of Indonesia's threatened species will not have been realized. Worse yet, REDD could effectively increase pressure to convert lowland mineral forest areas. This will severely limit biodiversity co-benefits of REDD in Indonesia, and risk undermining efforts to conserve biodiversity in the long-term, for three reasons.

First, overall biodiversity levels in peat forest are substantially lower than in lowland forest on mineral soils [81-83], reflecting the water-logged, nutrient-poor status and lower productivity of peat forests [84-86]. Peat forest plant diversity is less than half that of forest on mineral soils (Table 2; see Additional File 1: Datafile_1.xls for original data). Only 21 (15%) of Indonesia's 140 Critically Endangered lowland plant species have been recorded in peat, including three as specialists, compared to 104 (74%) found in lowland forest on mineral soils, 84 as specialists (Table 2; see Additional File 3: Datafile_3.xls for original data). Peat forests also harbour significantly fewer bat species (Table 2) and support lower densities of birds [107], bats and several keystone terrestrial and arboreal vertebrates, though not all (e.g. the orangutan, Table 2).

**Table 2:** Biodiversity attributes of lowland tropical forest (<500 m a.s.l.) on peat and mineral soil substrates in Sumatra and Kalimantan, Indonesia

	Lowland forest on contrasting substrates	
	
Taxon and Attribute	**Mineral soils**^** a**^	Peat
*(a) Woody plants*		

Species richness (number species per 100 stems) ^b^	35.2 ± 5.6 ***	15.1 ± 4.0

Fisher's alpha	80.9 ± 10.7 ***	18.2 ± 6.2

Critically Endangered species recorded present in forest on each substrate ^c^	114 recorded/84 specialists	21 recorded/3 specialists

*(b) Bats*		

Species richness (rarefied number species at standard sample) ^d^	16.6 ± 1.3 *	11.6 ± 0.8

Bat density (total abundance per trap night)	5.5 ± 2.7 *	2.0 ± 0.5

*(c) Densities of vertebrate species *^e^		

Water monitor *Varanus salvator*	0.22 ± 0.15 *	0.00 ± 0.00

Sun Bear *Helarctos malayanus*	0.06 ± 0.07	0.00 ± 0.00

Slow loris *Nycticebus coucang*	0.03 ± 0.06	0.00 ± 0.00

Helmeted hornbill *Rhinoplax vigil*	0.03 ± 0.06	0.00 ± 0.00

Small toothed palm civet *Arctogalidia trivirgata*	0.03 ± 0.06	0.00 ± 0.00

Pangolin *Viverra tangalunga*	0.03 ± 0.06	0.00 ± 0.00

Long tail macaque *Macaca fascicularis*	1.07 ± 0.41 ***	0.11 ± 0.16

Barking deer *Muntiacus muntjak*	0.62 ± 0.26 *	0.11 ± 0.16

Bushy crested hornbill *Anorrhinus galeritus*	0.47 ± 0.21 *	0.11 ± 0.16

Red leaf monkey *Presbytis rubicunda*	2.32 ± 0.54 ***	0.62 ± 0.47

Rhinoceros hornbill *Buceros rhinoceros*	0.45 ± 0.25	0.17 ± 0.19

Tufted ground squirrel *Rheithrosciurus macrotis*	0.20 ± 0.17	0.11 ± 0.16

Monitor lizard *Varanus *sp.	0.15 ± 0.13	0.11 ± 0.16

Bornean white bearded gibbon *Hylobates albibarbis*	3.68 ± 0.77	2.87 ± 0.87

Pale giant squirrel *Ratufa affinis*	0.65 ± 0.29	0.56 ± 0.34

Oriental pied hornbill *Anthracoceros albirostris*	0.59 ± 0.30	0.68 ± 0.40

Bornean bearded pig *Sus barbatus*	2.27 ± 0.58	2.65 ± 0.78

Bornean orangutan *Pongo pygmaeus*	0.98 ± 0.41	1.32 ± 0.65

Mouse deer *Tragulus *spp.	0.28 ± 0.17	0.39 ± 0.29

Pig tail macaque *Macaca nemestrina*	0.11 ± 0.11	0.17 ± 0.19

Prevost squirrel *Callosciurus prevostii*	0.17 ± 0.13	0.39 ± 0.29

Binturong *Arctictis binturong*	0.06 ± 0.08	0.17 ± 0.19

Wreathed hornbill *Aceros undulatus*	0.00 ± 0.00	0.06 ± 0.11

*(d) Densities of large vertebrate Orders*		

Artiodactyla (deer and pigs)	3.17 ± 0.71	3.15 ± 0.85

Primata (primates)	8.20 ± 1.40 *	5.09 ± 1.24

Bucerotidae (hornbills)	1.61 ± 0.51	1.01 ± 0.49

Carnivora (carnivores)	0.17 ± 0.14	0.16 ± 0.20

Comparisons are made between (a) woody plants, (b) bats, and (c & d) large vertebrates. Plant data compiled from published and unpublished literature, and mammal data are derived from field surveys. All data are mean ± 95% CI.

• *P *< 0.05, ** *P *< 0.01, *** *P *< 0.001

^a ^Forest on lowland mineral (non-swamp) soils excluding forest on limestone, ultrabasic rock and coarse textured sandy soil types on which kerangas vegetation develops.

^b ^Compilation of published and unpublished records of 'local scale' (defined as <3 ha total sample plot area) woody plant surveys for stems ≥10 cm diameter at breast height (see Additional File 1: Datafile_1.xls for original data; references [87-101]). The index 'Species per 100 stems' was computed as species per stem (total species number divided by total stem number) scaled to 100 stems. Total stem number per sample was similar between peat and mineral soils samples, 430 ± 328 vs 505 ± 265, respectively. Data compiled from n = 22 for peat and n = 24 for mineral areas. Richness and Fisher's alpha compared using two-tailed t-test adjusted for unequal variance.

^c ^Based on compilation of data on geographic range and habitat distributions from published and unpublished records for all IUCN-listed Critically Endangered (CR) plant species in Indonesia. Species shown as present in peat swamp forest are defined as all taxa with at least one record in forest reported as peat swamp forest. Species listed as present in forest on lowland mineral soils (non-swamp) are defined as all other CR species with records < 500 m a.s.l. minus those taxa that are considered specialists on azonal extreme geological features, including limestone, ultrabasic rock, or kerangas forest types that form on podzolized soils on coarse textured sedimentary rocks. Species treated as specialists on peat or mineral soils are defined as taxa with records from only one ecosystem type. A full accounting of CR species recorded as present in peat is provided in Additional File 2: Datafile_2.xls.

^d ^Based on harp-trap inventories of insectivorous bats captured at three locations each in forests on peat in Kalimantan (Danau Sentarum, Sungai Putri, Tanjung Puting), and forests on mineral soils in Kalimantan (Barito Ulu, Sungai Lesan) and Sabah (Danum Valley). Individual captures at each site were rarefied 1000 times in EstimateS to compare species richness at a standard number of individuals (n = 128, the capture number in the smallest inventory at Danau Sentarum). Capture rate - total bat abundance per trapping effort at a site - is a surrogate estimate of density.

^e ^Vertebrate densities were measured along permanent census routes in lowland forest on peat and mineral soil substrates at Gunung Palung National Park, West Kalimantan, Indonesia. Table shows total number of independent observations (Mean no. km-^2^) of large bodied vertebrates between August 2000-2002 in lowland mineral areas (N = 170 surveys, 591.7 km) and peat forest (N = 87 surveys, 290.6 km). Species are sorted by increasing relative density on peat versus mineral soils. Note that, as this is a sample from a single site, specific values as well as presence/absence may vary substantially across sites.

Second, biogeographically distinct sub-types of lowland forest on mineral soils are under-represented in Indonesia's protected area network [108,109], and many existing protected areas remain threatened by illegal logging, conversion to agriculture and fires [110,111].

Third, according to 2008 data, c. 5.4 million ha of remaining lowland mineral forest in Kalimantan (25% of the total) is zoned for conversion to non-forest agricultural uses, such as oil palm (Table 1). A further c. 12.4 million ha (58%) is zoned as production forest, which can be legally converted to fiber plantations. Combined, more than 80% of remaining species-rich lowland forest on mineral soils in Kalimantan (c. 17.8 million ha) is eligible for conversion.

There is a risk that preferential targeting of carbon-dense peatland under REDD will worsen long-term prospects for biodiversity conservation in Indonesia by intensifying pressures to establish plantations in forested mineral soil areas that offer lower emission reduction potential (Table 1) but support richer biodiversity and higher concentrations of threatened species (Table 2). This problem is not unique to Indonesia [19]. Similar unintended consequences from REDD could intensify pressure on relatively low-carbon, floristically-rich *cerrado *ecosystems suitable for soy expansion in Brazil, and logged forests throughout the tropics, which store less carbon, but not necessarily less biodiversity than their unlogged counterparts [112,113].

### Safeguarding biodiversity co-benefits of REDD

Despite meaningful progress made at COP 15 toward developing a REDD framework, it remains unclear whether and how biodiversity will be treated within REDD. A properly structured market mechanism could, in theory, promote more equal balance of REDD interventions across ecosystems with different biodiversity attributes and threat levels (see example of an auction based system in *8*). In the short-term, however, such an approach would likely gain traction only in voluntary carbon markets (e.g., Gold Standard emission credits of the CCBA carbon standard, [114]), and such markets are currently too limited to have global impact [115].

Instead, we believe that a regulatory approach will be required to ensure REDD delivers substantial long-term biodiversity co-benefits in tropical countries. We make three recommendations for regulation to be effective:

#### Recommendation 1

Countries must prepare their own explicit national targets for ecosystem and species protection across the full range of native ecosystem types and biogeographic sub-regions (where applicable). Where such plans already exist - for example, to meet commitments under the Convention on Biodiversity (CBD) - they must be re-evaluated, updated and revised in a transparent manner, preferably in accordance with methods approved by the UNFCCC (e.g. following [116]).

#### Recommendation 2

Using these targets, gap analyses should be conducted to identify ecosystem types currently under-represented in the protected area network (or within degraded protected areas that have lost their conservation value) and new areas required for priority species that have insufficient habitat to maintain large viable populations. Recent work by [109] for Sumatra provides a useful model to evaluate ecosystem representation.

#### Recommendation 3

With co-financing from REDD to offset opportunity costs of foregone (or restricted) development, results from the above can be used to redefine acceptable land-use practices within priority areas needed to fill biodiversity conservation gaps. Examples might include: (i) re-classifying land use status of forested areas slated for conversion to non-conversion forest uses; (ii) restricting silvicultural practices in specific production forest areas to reduce impacts and maintain high biodiversity value; or (iii) re-assigning forested areas of exceptional importance for strict protection as parks or nature reserves.

If such a national planning process were made a pre-requisite for multi-lateral and bi-lateral REDD funding, and REDD payments linked not only to verified emission reductions but also to biodiversity co-benefits, then net positive impacts on biodiversity would be ensured, and the negative potential impacts we describe would be reduced. A target-based approach also respects the sovereignty of countries to prepare their own targets, and fulfils objectives of the CBD, both for recipient (tropical) countries and donor (developed) nations who are signatories to the convention.

## Conclusion

Implementing REDD to optimize biodiversity co-benefits involves trade-offs with emissions reduction and cost. At a global scale, planning REDD interventions to meet biodiversity targets, rather than maximize avoided emissions, increases estimated cost only slightly [10]. Further study is required to understand cost impacts at sub-national scales where REDD will be implemented. Spatially explicit methods are being developed to make systematic comparison among alternative land use scenarios for meeting biodiversity targets [117] and can be readily adapted to incorporate emission reduction potentials or other socio-political targets [118].

Protecting tropical forests is a good idea for mitigating global climate change and conserving globally threatened biodiversity. The devil, however, is in the details: scientists, citizens and government must work closely to determine where REDD funds should be spent to achieve an acceptable balance between emission reductions from forest and enhanced long-term biodiversity conservation.

## Competing interests

The authors declare that they have no competing interests.

## Authors' contributions

GDP, PLW, MJS and AJM contributed and analyzed data, and wrote the manuscript; EM, KO and BY participated in writing and the development of ideas; AT, AR, AM, BP, NW, SH and LD contributed data on biomass, plant species, emissions and/or land cover; FS contributed biomass data and the computation of Fisher's alpha for plants. All authors read and approved the final manuscript.

## Supplementary Material

Additional file 1 **Species richness, biomass and emission parameters for lowland forest on peat and mineral soils in Indonesia, Brunei and Malaysia**. This file provides raw data and citations for information presented in tables and text of the manuscript comparing biodiversity, biomass and emission characteristics of lowland forest on peat and mineral soil substrates. Click here for file

Additional file 2 **Summary of REDD projects, programs and policy initiatives in Kalimantan and Sumatra, Indonesia**. This file provides a summary of REDD activities in Sumatra and Kalimantan, including name, location, supporting institution(s), approximate size (ha) of areas covered by the activities and substrate (peat or mineral soils). Click here for file

Additional file 3 **Summary of dipterocarp tree species recorded in lowland forest on peat soils in Sumatra, Kalimantan, Sarawak and Sabah, and their conservation status on the IUCN Red List**. This file provides a tabular summary of published and unpublished records for dipterocarp species recorded in at least one peat swamp forest site. Individual citations, conservation status under ICUN and some additional notes are provided for each species. Click here for file
