# Supplementary material for: Biodiversity Conservation in the REDD
Source: Carbon Balance Manag. 2010 Nov 23;5:7. doi: 10.1186/1750-0680-5-7 (PMC3002342; doi:10.1186/1750-0680-5-7)
Supplement: Additional file 2 — Summary of REDD projects, programs and policy initiatives in Kalimantan and Sumatra, Indonesia. This file provides a summary of REDD activities in Sumatra and Kalimantan, including name, location, supporting institution(s), approximate size (ha) of areas covered by the activities and substrate (peat or mineral soils). [file 1750-0680-5-7-S2.PDF]

Additional Data File 2.

Summary of projects, programs and policy initiatives under the Reducing Emissions from Deforestation and forest Degradation (REDD) framework taking place in (or with a policy focus on) Kalimantan and Sumatra, Indonesia. Summary is based on publicly available data obtained from the internet (as of 18 October 2010) and discussions with national and international organizations and individuals involved in REDD project development and policy initiatives in Indonesia. See explanatory notes at bottom of table.

Synopsis of main points: A total of 17 site-based REDD projects were recorded in Kalimantan and Sumatra. Of these 11 are entirely on peat, and six are in lowland forest on well drained mineral soils. The combined area covered by REDD projects in Kalimantan and Sumatra is 3.02 million ha, of which 1.69 million ha (56%) is on peat. Of the 1.33 million ha on mineral soils, more than half (750,000 ha) are concentrated in one project – the Ulu Massen avoided emissions project in Aceh – an area Gaveau et al. (2009) [Reference *71*] argue is at relatively low risk of deforestation due to steep terrain and geographic isolation. ***This suggests less than 600,000 ha of threatened lowland forest on mineral soils have drawn REDD project attention in Kalimantan and Sumatra***.

No.	Island	Project, Program or Policy intervention	Area (ha)	Description	Peat or Mineral	Participating companies, organizations or government agencies	Source(s)
**Kalimantan**							
1	K	Project	100,000	Conservation of the Upper Kapuas Lakes System	Peat	Fauna Flora International, Macquarie Bank	http://karbon-redd.blogspot.com/2009/08/redd-projects-in-indonesia.html
2	K	Project	57,000	Rehabilitation of the Sungai Putri peat swamp forest, Ketapang, Kalimantan	Peat	Fauna Flora International, Macquarie Bank	http://www.fauna-flora.org/news_kalimantan.php
3	K	Project	100,000	The Rimba Raya	Peat	Infinite Earth, Orangutan	http://www.infinite-earth.com/projects-

No.	Island	Project, Program or Policy intervention	Area (ha)	Description	Peat or Mineral	Participating companies, organizations or government agencies	Source(s)
				Biodiversity Reserve Project		Foundation International	details.html
4	K	Project	364,000	Mawas Peatland Conservation Area Project, Central Kalimantan	Peat	Orangutan Conservancy, The Dutch Royal Government, Shell Canada	http://forestclimatecenter.org/
5	K	Project	225,000	Katingan Conservation Area: A Global Peatland Capstone Project, Central Kalimantan	Peat	Starling Resources, Yayasan Puter, Universitas Palangkaraya, Terra Global Capital	http://starlingresources.com
6	K	Project	50,000	Sebangau National Park, Central Kalimantan	Peat	WWF, BOS, Wetland International, CARE	http://karbon-redd.blogspot.com/2009/08/redd-projects-in-indonesia.html
7	K	Project	100,000	Lamandau Wildlife Refuge, Central Kalimantan	Peat	Orangutan Foundation UK	http://forest-carbon.org/projects/project-locations/ecosystem-and-habitat-mapping-in-the-papuan-transfly-region
8	K	Sub-national Program	na	CKPP (Central Kalimantan Peatland Project)	Peat	CARE International-Indonesia, WWF-Indonesia, BOS-Mawas, the University Palangka Raya and Wetlands International.	http://ckpp.wetlands.org/
9	K	Sub-national program	3,163,000	Kutai Barat, HKM: Heart of Borneo; East Kalimantan	Peat & Mineral	WWF	http://www.wwf.or.id/berita_fakta/highlights/index.cfm?uNewsID=20105&uLangID=1
10	K	Sub-national	2,124,000	Berau, Indonesia	Peat &	TNC, ICRAF, Sekala,	http://www.nature.org/initiatives/climate

No.	Island	Project, Program or Policy intervention	Area (ha)	Description	Peat or Mineral	Participating companies, organizations or government agencies	Source(s)
		program		Climate Action Project; Kabupaten Berau Forest Carbon Program, East Kalimantan	Mineral	University Mulawarman, Winrock International, University of Queensland	change/work/art25992.html
11	K	Project	nd	West Kalimantan Community Carbon Pool	Peat & Mineral	Fauna Flora International	http://www.fauna-flora.org/REDD_kalimantan.php
12	K	Project	300,000	Malinau Avoided Deforestation Project	Mineral	Global Eco Rescue; Borneo Tropical Rainforest Foundation	http://karbon-redd.blogspot.com/2009/08/redd-projects-in-indonesia.html; http://www.eco-rescue.com/whatwedo
13	K	Project	54,000	Sumalindo: Heart of Borneo; East Kalimantan	Mineral	WWF	http://karbon-redd.blogspot.com/2009/08/redd-projects-in-indonesia.html
**Sumatra**							
14	S	Project	400,000	Kampar Ring Partnerships for Avoided Emissions Supporting Sustainable on Responsible Peatland Management, Riau	Peat	Leaf Carbon Ltd, APRIL/RAPP	http://karbon-redd.blogspot.com/2009/08/redd-projects-in-indonesia.html
15	S	Project	15,640	Kampar Carbon Reserve, Riau	Peat	APP & Carbon Conservation	http://www.redd-monitor.org/2010/10/05/carbon-conservation-gets-into-bed-with-asia-pulp-

No.	Island	Project, Program or Policy intervention	Area (ha)	Description	Peat or Mineral	Participating companies, organizations or government agencies	Source(s)
							and-paper-one-of-indonesias-biggest-forest-destroyers/#more-5933
16	S	Project	24,000	Protection of biodiversity through reduced deforestation (REDD) in the peatland forest of Merang; Southern Sumatra	Peat	GTZ, Ministry of Forestry, Local government	http://www.merang-redd.org/REDD/komponen-mrpp/pegelolaan-dan-perdagangankarbon.html
17	S	Project	250,000	Berbak Carbon Value Initiative; Jambi	Peat	ZSL/DEFRA	http://karbon-redd.blogspot.com/2009/08/redd-projects-in-indonesia.html
18	S	Project	101,000	Harapan Rainforest Project	Mineral	Burung Indonesia; The Royal Society for the Protection of Birds; Birdlife International	http://karbon-redd.blogspot.com/2009/08/redd-projects-in-indonesia.html
19	S	Project	750,000	Reducing Carbon Emissions from Deforestation in the Ulu Masen Ecosystem; Aceh	Mineral	Carbon Conservation and Provincial governor, Fauna Flora International	http://www.fauna-flora.org/redd_aceh.php
20	S	Project	75,000	Batang Toru REDD project; North Sumatra	Mineral	Yayasan Ekosistem Lestari, CI, District Govt., Various logging concession holders	
21	S	Project	50,000	Tesso Nilo Pilot Project – REDD; Riau	Mineral	WWF	http://karbon-redd.blogspot.com/2009/08/redd-projects-in-indonesia.html

No.	Island	Project, Program or Policy intervention	Area (ha)	Description	Peat or Mineral	Participating companies, organizations or government agencies	Source(s)
22	All	Policy	na	FORCLIME: Indonesian-German Cooperation Priority Area Climate Change	Program	GTZ, CIM, DED, InWEnt, KfW	http://www.forclime.org/
23	All	Policy	na	The LOI between Norway and Indonesia to reduce emissions from peatland and forest conversion	Peat & Mineral	Governments of Indonesia and Norway	

NOTES:

Project – A set of targeted actions and/or regulatory interventions applied over a specified area with clearly defined project boundaries aimed at reducing forest and/or land (peat) based emissions arising from planned or unplanned exploitation or other disturbance (e.g. protecting a clearly defined peat land area from being drained and converted to oil palm by purchasing the license and maintaining the area as forest).

Sub-national Program – A set of coordinated projects and/or policy engagements to be implemented across an area defined by sub-national political boundaries. It is anticipate that sub-national programs will be implemented as 'Model Nations', designed to experiment with (and overcome limitations on) technical, regulatory, social and financial dimensions of implementing REDD over large and heterogeneous areas. The development of monitoring, reporting and verification (MRV) methods will be a major focus. Like projects, sub-national programs have clearly defined boundaries, but such boundaries correspond to Provincial or Regency level administrative units with authority over the program area. Unlike projects, however, program activities will be implemented across a sub-set of the program area with policy or on-the-ground interventions defined by specific projects. In Indonesia, most sub-national programs are still in early stages of development, with

specific intervention activities and policy changes to be defined. As such, it is not possible to deduce the extent to which peat versus mineral soil areas will be affected by activities of a REDD program at this time.

Policy – A national scale initiative, typically forming a government to government policy dialogue designed to review and revise land use planning and/or natural resource management decisions with the specific aim of reducing forest and/or land (peat) based emissions. The policy engagement may or may not include financial incentives in the form of monetary compensation paid for implementation of specific emission reduction activities. Agreements forged as part of such policy dialogues are likely to be implemented either as coordinated sub-national programs (e.g. across a province) or as autonomous projects developed in response respond to market or policy incentives created by governments.

nd = No data available
na = Not applicable

K = Kalimantan
S = Sumatra
All = Indonesia
